# Assessing patients’ attitudes to opt-out HIV rapid screening in community dental clinics: a cross-sectional Canadian experience

**DOI:** 10.1186/s13104-016-2067-6

**Published:** 2016-05-10

**Authors:** Mario Brondani, Steve Chang, Leeann Donnelly

**Affiliations:** Division of Preventive and Community Dentistry, Department of Oral Health Sciences, Faculty of Dentistry, University of British Columbia, 2199 Wesbrook Mall, Vancouver, BC V6T 1Z3 Canada; Surrey, BC Canada; Department of Oral and Biomedical Sciences, Dental Hygiene Program, Faculty of Dentistry, University of British Columbia, Vancouver, BC V6T 1Z3 Canada

**Keywords:** HIV, Screening, Patient care, Diagnosis, Dental setting, Patient’s perspective, Quantitate research, Survey

## Abstract

**Background:**

As a public health initiative, provided-initiated HIV screening test in dental settings has long been available in the U.S.; it was only in 2011 that such setting was used in Canada. The objective of this paper was to assess patients’ response to, and attitudes towards, an opt-out rapid HIV screening test in a dental setting in Vancouver, Canada.

**Methods:**

A cross-sectional evaluation design using a self-complete survey questionnaire on self-perceived values and benefits of an opt-out rapid HIV screening was employed. An anonymous 10-item questionnaire was developed to explore reasons for accepting or declining the HIV rapid screening test, and barriers and facilitators for the HIV screening in dental settings. Eligible participants were male and female older than 19 years attending community dental clinics and who were offered the HIV screening test between June 2010 and February 2015.

**Results:**

From the 1552 age-eligible patients, 519 completed the survey and 155 (10 %) accepted the HIV screening due to its convenience, and/or free cost, and/or instant results. From the 458 respondents who did not accept the screening, 362 (79 %) were between the ages of 25 and 45 years; 246 (53.7 %) had identifiable risk factors for contracting HIV; and 189 (41.3 %) reported having been tested within the last 3 months. Those tested in less than 3 months had 3.5 times higher odds to decline the HIV screening compared to those who have been tested between 3 months and 1 year.

**Conclusions:**

Convenience, cost-free and readily available results are factors influencing rapid HIV screening uptake. Although dental settings remain an alternative venue for HIV screening from the patients’ perspectives, dental hygiene settings might offer a better option.

## Background

HIV is a retrovirus that mainly attacks CD4 cells, leading to a progressive deterioration of the immune system [1]. There are approximately 72,000 Canadians living with HIV and it is estimated that one out of five of these individuals are unaware of their status and remain infectious [2, 3]. As a public health initiative, earlier diagnosis and access to treatment remains crucial for reducing the rates of transmission and optimizing HIV medication uptake [4, 5]. In fact, early diagnosis equates to a healthier life, offers a more favorable response to therapies, is cost-effective over the long term, and can reduce HIV transmission because more than 50 % of new HIV infections are believed to be transmitted by individuals unaware of their serostatus [6, 7]. In an attempt to reach those 25 % of potentially infectious individuals, efforts have been put forward to advocate for voluntary HIV opt-out screening point-of-care in alternative settings routinely for all individuals, and particularly for those engaged in high-risk behaviors [1, 8]. As a result, a shift in public policy took place in 2006 in the United States (U.S.) when the Centers for Disease Control and Prevention (CDC) recommended an HIV screening test to be offered by various health care providers to all individuals, including dental patients as seen worldwide [9–15]. In Canada, similar recommendations and guidelines were put forth a few years later by the Public Health Agency of Canada [2] and in British Columbia [3].

There are currently two forms of HIV rapid screening available with slightly different sensitivity and specificity, both looking for HIV antibodies: one collects oral fluids via a swab (92 % sensitivity and 99.98 % specificity at a 95 % confidence interval) [16], while the other uses whole blood via a finger-prick (99.8 % sensitivity and 99.5 % specificity at a 95 % confidence interval) [17]. Both tests are easy to administer and the U.S. Preventive Services Taskforce grades the screening for HIV an ‘A’ given its substantial net benefit for optimizing earlier HIV diagnosis for adolescents, adults, and pregnant women [18]. If the screening test shows a non-negative result, venipuncture is required to confirm the infection. Since 2006, dental clinics across the U.S. have been offering the screening test with a varied level of acceptance to the test by dental professionals and patients [15–18]. In 2011, we developed a partnership with the seek and Treat for Optimal Prevention of HIV/AIDS (S.T.O.P.) program and with the ‘*Does HIV Look Like Me?*’ International Society in Vancouver, Canada. This partnership was aimed at piloting a seven-month project to introduce routine dental provider-initiated HIV screening in community dental clinics [19]; this was the first time that such a setting was used in Canada.

This Canadian initiative emerged from a partnership between academia and the public health care system which brought together the authors, public health physicians and nurses, and not-for-profit organizations. The S.T.O.P program has been proposed to expand access to HIV/AIDS testing and medications in Vancouver and Prince George [24]. The implementation of the S.T.O.P Program also targeted those individuals facing multiple barriers to care, including those with history of addiction, mental health issues, homelessness and other social and environmental health determinant factors. More specifically, the S.T.O.P project aimed to:

- Ensure timely access to high-quality and safe HIV/AIDS treatment;
- Reduce the number of new HIV infections;
- Reduce the impact of HIV/AIDS through effective screening and early detection;
- Improve the patient experience in every step of the HIV/AIDS journey, from diagnosis to treatment;
- Improve the efficiency and cost-effectiveness of HIV/AIDS service delivery by linking patients directly to proper care and antiretroviral medication which decrease mortality and costly hospitalization.

The pilot initiative ran a seven-month project, from June 2011 to January 2012, to introduce an opt-out routine dental provider initiated screening for HIV infection as an element in a standard dental examination, along with x-rays, charting for tooth decay and periodontal disease, and a head and neck examination. The HIV Screening in Dental Clinics pilot project trained 10 dental professionals (including dentists, dental hygienists, and certified dental assistants) from three different community dental clinics on pre- and post-counselling, ongoing psychosocial support and education. After the training, however, only two dental community clinics decided to offer the HIV screening once a week, and they continued to offer the test after the pilot phase was over; the other clinic opted voluntarily to not offering the HIV screening to its clients. The rationale for targeting dental clinics relies on the allegedly higher frequency that people may see a dentist compared to another type of health care provider. In fact, in 2011 the Canadian Health Measure Survey released its vast amount of demographic, behavioural and clinical health data which was collected through household interviews and direct physical measures at mobile examination centres with a 97 % representation of the Canadian population aged 6–79 years old [20]; dental care seeking behaviour tend to be preventive-driven. According to the CHMS, many Canadians seem to not access primary care physicians because they do not feel “sick” or show any symptoms of (any) illness; medical care seeking behaviour tend to be symptom-driven. Likewise, people may not express any signs or symptoms of HIV infection over the first few weeks as the initial infection produces nothing more than a mild disease that is self-limiting; about 30 % of infected patients remain asymptomatic during that same period. As a result these patients might not seek any primary care and many do not even have a regular primary care provider. By contrast, dentists may see these individuals more consistently to the extent that approximately 64 % of Canadians older than 12 years had visited a dental office once a year and 50 % of these individuals visited their dentists twice a year or more. Dental and oral care have been an ally to HIV primary care since the early 1980s, when virtually all HIV-positive patients could present with an oral manifestation in the form of opportunist infection related to progression of the HIV disease [21–24]. Moreover, dental care providers see the benefit in providing HIV screening in dental settings [25, 26] as they are often the first with the opportunity to recognize symptoms consistent with HIV infection that takes place in the mouth [27, 28]; the opportunity to offer the HIV screening cannot be lost.

In the meantime in the U.S., 3.5 million individuals who have been identified as at high risk for HIV infection have not been offered an HIV test in the past 5 years, while 75 % of them have visited their dentist with in the past 2 years [30]. Missed opportunities for HIV diagnosis abound and infections continue to occur. Various studies have shown that the scope of practice, skills and training, patient reactions [29] and logistics might be perceived barriers for fully implementing the HIV rapid testing in dental-related settings [28–30]. Nonetheless, worldwide studies have also shown that, in general, dental professionals and patients seem to consider chair-side screening for HIV as an important component of dental care [16, 17, 28, 31, 41]. It remains unknown what the level of acceptance is for, and the barriers in implementing, HIV rapid screening in Canadian dental settings. This study had the following objectives: to assess patient response to the incorporation of an opt-out rapid HIV screening test in dental appointments; to determine patient attitude towards dentists performing HIV screening, and to identify barriers in offering rapid HIV screening in dental settings from the patients’ perspective.

**Fig. 1:**
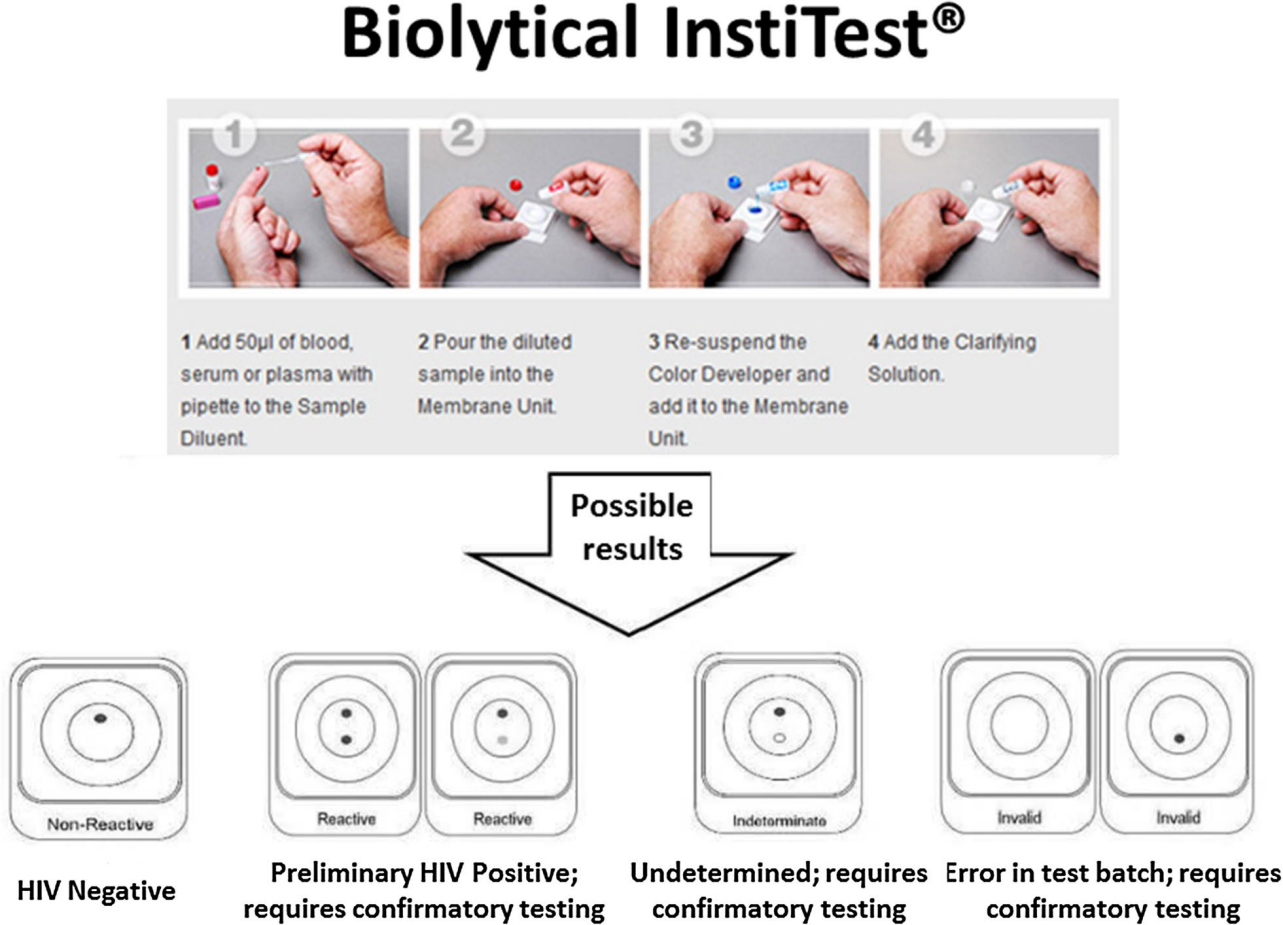
Finger prick Rapid HIV test used in this study (bioLytical INSTI™)

A pilot study [31] as well as ongoing initiatives have introduced the free-of-charge, finger-prick HIV screening (Fig. 1) in two local dental community clinics in Vancouver. One of the clinics is located at the MidMain Community Health Center [30], the largest not-for-profit clinic in the province that provides comprehensive health and oral care to an average of 4000 patients a month including refugees, new immigrants, Aboriginal people, as well as Caucasian and South-East Asian communities. Another clinic is tied with the University of British Columbia, Faculty of Dentistry General Practice Residency program that takes place at different locations across the greater Vancouver and lower mainland areas. The Residency program involves graduated dentists and their preceptors, some of which were trained on the HIV rapid testing and pre- and post-counselling. The residents are on a rotation schedule under supervision.

## Methods

As presented elsewhere [31], an anonymous 10-item self-complete questionnaire was developed in English to assess the reasons for having the HIV rapid screening test (e.g., convenience, and/or free cost, and/or speedy results) or not (e.g., unprepared, and/or uncomfortable setting, and/or not at risk for HIV infection); to determine patient attitude towards dentists performing HIV screening (e.g., as part of the regular dental check-up appointment, and/or convenience of the dental chair, and/or as within the dental professional scope of practice); and to identify barriers in offering HIV rapid screening in a dental setting (e.g., not within the scope of practice, and/or not wanting the finger poked, and/or unprepared, and/or not comfortable in the dental setting). The survey did not ask for respondents’ educational levels, financial status, or ethnicity.

Since the survey questionnaire was administered within the HIV rapid screening initiative, it seems worthwhile to note that the test was offered as the medical and dental histories were being taken, and before any clinical procedure was performed. The option for the HIV screening was given to all patients who were receiving dental and dental hygiene care, and who were older than 19 years, HIV negative or of unknown status, and of any gender and sexual orientation. The questionnaire was also offered to all patients independently of any exposure to risk factors for HIV infection as per their medical history. In summary, the inclusion criteria for this study enrolled all the patients independently of having accepted the HIV rapid screening test or not, between June 2010 and February 2015 (as a cut off timeframe including the pilot, and despite the HIV rapid testing being offered continuously). Exclusion criteria comprised of those patients who declined to answer the survey, whether or not they have been screened for HIV. The option for the questionnaire survey was then given by dental professionals to all eligible patients at the conclusion of the dental appointment as they exited the dental operatory. Participants were advised that the survey was totally voluntary and anonymous, would take less than 5 min to complete, had no barrels to their current and future dental appointments, and could be completed in the waiting area before they left the clinical setting. If they decided to complete it, they were advised to remove the cover page of the survey as proof of their consent to the study and to publish, and place the one-page questionnaire survey in a cardboard box which was placed by the front-desk of the clinics where the front desk personnel could ensure its security. The surveys were collected weekly by the authors at the end of the Friday’s shift at 5:00 PM. Neither the authors nor the front desk staff kept track of which participants completed the survey and which did not.

### Ethics, consent and permissions

The University of British Columbia ethical approval was obtained (#H11-02138), and participants voluntarily consent to take part in this study.

### Consent to publish

Consent to publish was obtained from the participants as they retained the cover page of the survey showing to following statement: *By removing this cover page, you indicate that you consent to participate in this study. You have given permission for the principal investigators to use the information you are providing anonymously as part of a publication focused on the same issue.*

### Statistical analysis

Data was inputted into SPPS^®^ 21 (IBM^®^ Corporation, Somers, NY, USA) and standard descriptive statistics were calculated. Comparisons were made between demographic data (e.g., sexual orientation, gender, history of IV drug use) and whether HIV screening was accepted or rejected, reasons for accepting or rejecting the screening, willingness to get tested in a medical setting or at the next dental visit, and so on. Multivariate logistic regression was performed and odds ratio (OR) with 95 % confidence interval (95 % CI) are presented.

## Results

Between June 2010 and February 2015, there were a total of 1552 age-eligible patients seen who were offered the HIV rapid screening test and the survey questionnaire. Of these, 155 accepted being screened (10 % out of 1552 age-eligible patients) and they were all negative as per the test results; sixty-one HIV screened patients also completed the survey questionnaire. From the patients who declined to get screened, 458 answered the survey for a total of 519 respondents (33.4 % from 1552); 370 were males and between the ages of 19 and 85 years (Table 1).

**Table 1:** Demographic characteristics of 519 patients completing the survey about an opt-out HIV rapid testing in a dental clinic

Participants	Number (%)
Male	370 (71)
Female	149 (29)
Accepting the HIV screening	61 (12)
Declining the HIV screening	458 (88)
Age distribution (years)	
19–24	78 (15)
25–44	384 (74)
≥45	57 (11)
Risk factors (not mutually exclusive)	
MSM^a^	92 (18)
Unprotected sexual activities in the past 3 months^b^	189 (36.5)
Intravenous drug use^b^	12 (2.5)
Unprotected sexual activities in the past 3 months + intravenous drug use	70 (13)
No identifiable risk factor	156 (30)

^a^Men who have sex with men

^b^Yes/no

Among the 61 participants who were both screened and completed the survey, 44 were females (72 % out of 61 participants) and 25 (41 %; 19 males and six females) reported having at least one identifiable risk of contracting HIV: unprotected sexual activities in the past 3 months and/or use of intravenous drugs. When asked ‘*why did you choose to have the test?*’, all 61 respondents reported that they accepted the HIV screening due to its convenience, and/or being free-of-charge, and/or being able to receive the results on the spot (respondents could select more than one option). Almost 70 % of these 61 respondents would like to have the HIV rapid screening as part of a regular dental appointment while 93 % felt that dental offices were appropriate venues for HIV screening. Fifty-seven respondents would recommend the HIV screening in this dental setting to their peers. Interestingly, 11 (18 % out of 61 respondents) reported being screened in the past 3 months, and yet accepted the screening this time.

Among the 458 respondents who completed the survey but declined the HIV screening, 362 (79 %) were between the ages of 25 and 45 years while 92 self-identified as men-who-have-sex-with-men. Two-hundred and forty-six (53.7 % out of 458 respondents) had identifiable risk factors for contracting HIV: 189 had unprotected sexual activities in the last 3 months, 45 had unprotected sexual activities in the last 3 months and were intravenous drug users and 12 were intravenous drug users. When asked the reasons for not getting screened, 45 % (206 out of 458 respondents) reported having been screened within the last 3 months, 24 % (110 out of 458 respondents) felt that they were not at risk of contracting HIV, 15.5 % (71 out of 458) did not want to have their finger poked, 8.9 % (41 out of 458 respondents) reported having been screened more than 3 months ago, and 7.2 % (33 out of 458 respondents) were not prepared to have an HIV screening that day. Two hundred and fifty-one were never screened for HIV. Three-hundred and forty four (75.1 %) of those that declined the test felt that HIV screening was still within the scope of practice of a dentist (*strongly agree* and *agree* answers combined), 101 (22 % out of 458 respondents) would have been tested if the test had been offered by a family physician, and 69 (15 % out of 458 respondents) would have accepted the test if it was an oral swab. All of these 69 respondents favoring an oral swab HIV screening were among the 71 who reported not wanting to have their finger poked. Ten respondents (2.2 % out of 458 respondents) were willing to get screened in the next dental visit. From the 151 respondents who reported not being at risk of contracting HIV (110 participants) but having been tested within the last 3 months (41 participants), almost 30 % of them have had unprotected sex in the past 3 months.

Table 2 shows the contribution of gender to agreement levels for the statement that HIV screening should be part of dental care (*strongly agree* to *strongly disagree*). Note that among those who strongly agreed or agreed with that statement, 67.5 % (from 444 who *agreed *or *strongly agreed*) were males while among those who disagreed or strongly disagreed, 69.3 % (from 75 who *disagreed* or *strongly disagreed*) were males.

**Table 2:** Distribution of agreement levels of 519 respondents according to gender as per the statement ‘diagnosing HIV is part of a dentists’ job

Gender	SA (n = 268 %)	A (n = 176 %)	D (n = 62 %)	SD (n = 13 %)	*P* value^a^
HIV screening is part of dental care					
Male (n = 270)	176 (65.6)	124 (70.4)	41 (66.2)	11 (84.6)	<0.01
Female (n = 128)	92 (34.4)	52 (29.6)	21 (33.8)	2 (15.4)	

*SA* strongly agree, *A* agree, *D* disagree, *SD* strongly disagree

^a^ *P* value corresponds to the test of liner trend for categorical data using the Cochran-Mantel–Haenszel Chi square test

Table 3 shows the multivariable logistic regression analysis of HIV screening uptake. The odds of declining the screening test was 2.4 times higher for those who had been tested in less than 3 months compared to those who tested in more than 3 months, keeping the other variables constant. The regression analysis also revealed that females more than males had higher odds to accept the test (OR 2.36, CI 2.09–3.01) while those with one or more identifiable risk for HIV infection had 1.88 higher chances to decline the test compared to those who self-perceived being not at risk for HIV infection, keeping all the other variables constant.

**Table 3:** Estimated unadjusted odds ratios (OR) using multivariate logistic regression analysis HIV screening test

	HIV screening Declining N (%)	HIV screening Accepting N (%)	OR^b^ (95 % CI)	*P* value
Gender	*364 (70)*	*155 (30)*		
Male	280 (77)	93 (60)	0.89 (0.69–1.08)	0.215
Female	84 (23)	62 (40)	2.36 (2.09–3.01)	0.047
Being tested previously^a^	*223 (70)*	*95 (30)*		
Less than 3 months	100 (44.8)	25 (26.3)	2.42 (2.11–2.87)	0.001
More than 3 months	123 (55.2)	70 (73.4)	0.93 (0.73–1.01)	0.109
Identifiable risk for HIV infection^c^	*364 (70)*	*155 (30)*		
Yes	250 (68.7)	97 (62.6)	1.88 (1.19–2.11)	0.060
No	114 (31.3)	58 (37.4)	0.94 (0.77–0.99)	0.271

Data from this table refers to the 519 respondents who completed the questionnaire

^a^The data refers only to those who reported being screened for HIV at any time in their lives. 201 were never screened for HIV and are not shown

^b^The reference group was ‘HIV screening—declining’. The coefficient estimated indicated likelihood of accepting the HIV screening

^c^Included all 519 respondents including those who reported having had unprotected intercourse and/or using of intravenous drugs for the past 3 months

## Discussion

From the 1552 age-eligible patients who were offered the HIV rapid screening test, 155 accepted to be screened, or about 10 %. Studies in the U.S. evaluating opt-out HIV rapid screening testing in dental settings have reported acceptance rates above 70 % as found by Hutchinson and colleagues [26] and others [31], more than sevenfold higher compared to the findings from this study. However, contrary to our study, Blackstock and colleagues [31] hired a fully-trained counselor to screen patients as they waited for the dental appointment for a 97 % uptake, while Dietz et al. [32] focused on the intention to get tested but with no patients actually getting screened. The acceptance rate of 10 % seen in our study is also lower compared to the average uptake of 50 % in emergency departments and sexually transmitted infection clinics [33, 34]. These venues, contrary to a dental-related setting, do focus on HIV screening as paramount to their treatment delivery system with sexually transmitted infections, which is not the primary reason that people go to a dental clinic.

The majority of the respondents who got screened for HIV (70 % out of 61 participants) and of those who declined to be screened for HIV (75 % out of 458 participants) felt that such initiative was still within the scope of practice of a dentist, which corroborates Greenberg and colleges [41]. Among those who declined the test, 200 have been screened more than 3 months ago; those who got tested in less than 3 months were 2.4 times more likely to decline the HIV screening compared to those who have been tested more than 3 months ago, although improvements in HIV test window period have made the 3 months wait unnecessary [35]. Nonetheless, it remains a bit of a concern that 53.7 % of our participants who declined the HIV screening had one or more identifiable risk factors for being potentially exposed to HIV. In fact, the results from this study seemed to show that some people might still hold misconceptions about risks for HIV infection as advised by Milaszewski’s team [36]. Those who reported having unprotected sexual activities in the past 3 months did not think they were at risk for HIV infection, as also discussed by Balan and colleagues [37]. Respondents who had unprotected sexual activities in the past 3 months were as likely to decline the HIV screening as those who self-perceived being not at risk for HIV infection (data not shown). Such findings have to be interpreted with caution, however. Eleven of these participants wrote down voluntarily in the questionnaire that they were in a monogamous (either same or opposite sex) relationship for which sexual protection might not apply. Given the constraints of a questionnaire survey used, issues such as tested-related privacy, psychosocial support and linkage to medical care might have been potential factors influencing test uptake as found by VanDevanter and co-workers [38]. However, these factors were not explored in this study.

As per the questionnaire response rate, 33.4 % (519 out of 1552) can be considered a fair enrolment, in line with other studies administering anonymous and volunteer surveys as found by Brondani [39] and others [40]. Moreover, the context in which the questionnaire was administered, e.g., after a dental appointment that can be stressful to many patients, might have prevented people from completing it as they would rather leave the clinic and not ‘hang around’. All respondents who accepted the HIV rapid screening test reported doing so due to its convenience, and/or no cost and/or instant results. These were the same advantages identified in a qualitative interview study with patients conducted by VanDevanter and colleagues in 2012 [38], even though patients were interviewed about an oral swab HIV rapid screening test, which is less invasive than the finger-prick version used in our study.

The survey questionnaire presented here is part of a study to introduce provider initiated opt-out HIV rapid screening tests in dental settings in Vancouver, Canada. As a provider initiative, all age-eligible patients were offered the HIV screening independent of their gender, sexual orientation, socioeconomic status, ethnicity and any other qualifier. As a result, this study cannot be compared to those by Freeman and colleagues [34] who found that males were less likely to be approached for the HIV test than females. Hutchinson and coworkers [26] found that dental faculty and students considered scope of practice, training and patient reaction as factors influencing the successful implementation of the HIV rapid screening test in dental settings. Although this current study included only patients, they similarly recognized that such a procedure does fall within the scope of dental practice.

One of the potential barriers found to prevent some of our respondents from accepting the rapid HIV screening seemed to be the mode of administration: 15 % out of 458 respondents who declined the HIV screening did not want their finger poked, but would have consented to get screened for HIV if an oral swab procedure was offered instead, which is not currently available in Canada. Although Donnell-Fink and colleagues [41] did not find a significant difference in acceptance rates when comparing finger-prick with oral swab tests, their participants were not given the option and were randomly allocated into two groups, respectively. Nonetheless, Health Canada has only approved the finger-prick version shown in Fig. 1 at this point, e.g., April 2016.

The major strength of this study include being part of an initiative that introduced a provider initiated opt-out HIV rapid screening test as point of care in dental settings following a successful pilot initiative in Vancouver, Canada [31]. Another strength was the fact that we offered patients the opportunity to evaluate the HIV rapid screening test itself, its feasibility in a dental setting, and to identify some of the barriers and facilitators for testing uptake. However, further studies are needed to expand on the findings, to enroll a larger sample from various locations, and to fully evaluate the use of HIV screening test in alternative dental-related locations including community settings and engaging other professionals such as dental hygienists. In fact, this initiative has now been expanded to include routine HIV screening at dental hygiene educational settings via the Faculty of Dentistry at the University of British Columbia. Although such settings are currently being evaluated, Brondani and Chang [16] highlighted that without buy-in from the dental profession at large, little can be done to successfully implement HIV rapid screening in any dental settings; recent efforts to reach out the dental profession put forward by the Canadian Dental Association is a positive step [42]. Hence, full acceptance of such screening practice also requires an attitudinal change by the public.

## Conclusions

As we asked ourselves ‘*will patients be receptive to point of care HIV testing as a part of routine dental care?*’ [31], we can say that almost 10 % of all eligible patients did opt to get tested due to its convenience, free of cost and instant results. From the 519 respondents to the survey, 206 reported having been screened within the last 3 months while 246 had at least one identifiable risk for contracting HIV. Although the few patients who had consented to the test prevents generalizations of this study and the lack of other Canadian studies limits national comparisons, the idea of optimizing HIV rapid testing in dental settings is still new in Canada and not fully developed as a mainstream activity. HIV rapid screening test remains promising for reaching a proportion of the population that is not accessing conventional primary health care, but visit their dental professionals more regularly. The several challenges and barriers to implement HIV testing require a wide spread training of the dental team; availability of less invasive HIV rapid testing in Canada; and a mind-set change from the public who might be reluctant in associating HIV testing with dental appointments of any kind.

From a health promoting public health stand-point, HIV screening in clinical dental settings can benefit the Canadian population once it is provided following best-practice guidelines and with support for proper medical care and counseling when necessary. We urge the profession as well as other national and worldwide dental schools to come together to continue the conversation around HIV rapid testing in alternative health care settings like dentistry.
